# Pediatric appendicitis rupture rate: a national indicator of disparities in healthcare access

**DOI:** 10.1186/1478-7954-3-4

**Published:** 2005-05-04

**Authors:** Kathleen A Jablonski, Mark F Guagliardo

**Affiliations:** 1 The Biostatistics Center, The George Washington University, 6110 Executive Boulevard, Suite 750, Rockville, Maryland 20852, USA; 2 Department of Prevention and Community Health, The George Washington University School of Public Health and Health Services, Washington, DC, USA; 3 Center for Health Services and Community Research, Children's National Medical Center, 111 Michigan Avenue, NW, Washington, DC 20010, USA

## Abstract

**Background:**

The U.S. National Healthcare Disparities Report is a recent effort to measure and monitor racial and ethnic disparities in health and healthcare. The Report is a work in progress and includes few indicators specific to children. An indicator worthy of consideration is racial/ethnic differences in the rate of bad outcomes for pediatric acute appendicitis. Bad outcomes for this condition are indicative of poor access to healthcare, which is amenable to social and healthcare policy changes.

**Methods:**

We analyzed the KID Inpatient Database, a nationally representative sample of pediatric hospitalization, to compare rates of appendicitis rupture between white, African American, Hispanic and Asian children. We ran weighted logistic regression models to obtain national estimates of relative odds of rupture rate for the four groups, adjusted for developmental, biological, socioeconomic, health services and hospital factors that might influence disease outcome.

**Results:**

Rupture was a much more burdensome outcome than timely surgery and rupture avoidance. Rupture cases had 97% higher hospital charges and 175% longer hospital stays than non-rupture cases on average. These burdens disproportionately affected minority children, who had 24% – 38% higher odds of appendicitis rupture than white children, adjusting for age and gender. These differences were reduced, but remained significant after adjusting for other factors.

**Conclusion:**

The racial/ethnic disparities in pediatric appendicitis outcome are large and are preventable with timely diagnosis and surgery for all children. Furthermore, estimating this disparity using the KID survey is a relatively straightforward process. Therefore pediatric appendicitis rupture rate is a good candidate for inclusion in the National Healthcare Disparities Report. As with most other health and healthcare disparities, efforts to reduce disparities in income, wealth and access to care will most likely improve the odds of favorable outcome for this condition as well.

## Background

The persistence of racial and ethnic disparities in health and healthcare is a major theme in American healthcare policy. The Institute of Medicine report, "Unequal Treatment: Confronting Racial and Ethnic Disparities in Health Care" [1], describes and underscores the problem, and Healthy People 2010 [2] has made disparity elimination one of its two overarching goals. In response to these concerns Congress directed the Agency for Healthcare Research and Quality (AHRQ) to prepare a National Healthcare Disparities Report [3,4]. to monitor progress toward alleviating disparities. Over 250 measures of healthcare quality and access were considered for the report. However, while many measures are relevant to children, only 16 are specific to children. Of these, only two concerned pediatric inpatient services. Therefore the National Report could be improved by the inclusions of additional markers of pediatric health and healthcare disparities.

Appendicitis outcome is a good candidate measure because it is the most common intraabdominal surgical procedure performed on children [5], and may be the most common of any surgery among adolescents [6]. Furthermore, it has no known links to behavioral or social risk factors, and has only one treatment option – appendectomy. Timely surgery within a few hours or days of symptom onset is necessary to prevent rupture and other complications, which are costly [7] can result in loss of internal organs, female infertility and even death [8].

Therefore it is disconcerting that racial and ethnic disparities in the rate of appendicitis rupture (AR) have recently been reported among children of California and New York [9]. Compared to white children, odds of rupture was elevated by as much as 47% for African American children, 45% for Hispanic children, and 116% for Asian American children, after adjustments for the biologic risk factors, age and gender. Disparities were ameliorated but still apparent after adjustments for income, insurance status, and hospital characteristics. Subsequently, Ponsky et al. [10] analyzed a large sample of pediatric appendectomy cases from free-standing U.S. children's hospitals. Although their data lacked an indicator for Hispanic ethnicity, they found that odds of rupture was 16% higher for African Americans and 66% higher for Asians compared to whites.

While groundbreaking, neither previous study used a nationally representative sample, which limits their usefulness for National Healthcare Disparities Report. However, an underutilized federal survey, AHRQ's Healthcare Cost and Utilization Project (HCUP) Kids' Inpatient Database (KID), can be used for the purpose. KID is a stratified national sampling of pediatric discharges. Its latest version, from 2000, contains 2,516,833 discharges from 2,784 hospitals, weighted to represent 7,291,032 pediatric discharges nationwide. Therefore, KID is uniquely qualified to answer questions about pediatric inpatient services, including emergency surgical services, at the national level. In this paper we will report the latest estimates for frequency of pediatric acute appendicitis, and AR rates among all U.S. children, and determine if the previously reported disparities in AR rates are apparent at the national level. If KID bears evidence of disparities in AR rates, then this information should be included in the National Report.

## Methods

### Data source

This was a retrospective cohort study of the Kid's Inpatient Database (KID) 2000, a member of AHRQ's Healthcare Cost and Utilization Project (HCUP) family of databases [11]. The database was specifically designed to allow researchers to track and analyze national trends in pediatric inpatient utilization, outcomes and quality. KID 2000 contains 2,516,833 abstracts representing a finite population of 7,291,039 children derived from the pediatric discharges of 2,784 hospitals in 27 states during the calendar year 2000. The sampling frame includes all pediatric discharges from community, non-rehabilitation hospitals in the HCUP State Inpatient Databases (SID) that could be matched to American Hospital Association survey data. It is from the latter that KID derives hospital characteristics for each discharge. Case weights were based on stratification on six hospital characteristics. Admission age was <21 years for all cases. The sampling procedure selected 80 percent of non-birth pediatric discharges from each hospital in the sampling frame. The sampling design and weighting details are described elsewhere [11].

### Cases

We followed the methods of Guagliardo et al. [9] for identifying non-incidental pediatric appendicitis cases and cases with rupture or complications. Cases were limited to children 4 to 18 years old because acute appendicitis is rarely diagnosed in a timely manner for very young children, regardless of race/ethnicity or access to care considerations [12]. Appendicitis cases were defined as any discharge with any ICD-9 CM diagnosis code in the 540.X range. Cases were excluded if an appendectomy was incidental (ICD-9 CM procedure code 47.1X) or there were comorbid conditions likely to hinder the timely diagnosis of appendicitis, such as injuries to the GI tract, GI neoplasms, or inflammatory bowel disease. (A complete list is available from the authors upon request.)

Within this sample, cases with rupture or other complications were identified as having any of the following ICD-9 CM diagnosis codes: 540.0 (acute appendicitis with generalized peritonitis), 540.1 (acute appendicitis with peritoneal abscess), 567.2 (other suppurative peritonitis), 569.5 (abscess of intestine), 614.3 (acute parametritis and pelvic cellulitis), and 614.4 (chronic or unspecified parametritis and pelvic cellulitis). Guagliardo et al. [9] also classified appendicitis cases with codes 682.2 (other cellulitis and abscess-trunk) and 998.59 (other postoperative infection) as having complications. However we agree with the reviewers and editors of this journal that these conditions could arise post-operatively in both complicated and non-complicated appendicitis cases. Therefore we removed from all analyses the 73 cases with either 682.2 or 998.59 that did not otherwise qualify for inclusion. To remain consistent with previous reports we refer to all cases with rupture or other complications as "rupture cases", although technically all were not ruptured.

Finally, we excluded cases with race/ethnicity designations that were missing, Native American, or "Other", as there were too few of these cases for meaningful analysis. This left us with a final sample of 32,784 cases, representing a weighted count of 62,555 patients.

### Variables

#### Main predictor

Race is coded in the KID 2000 database as white, black, Hispanic, Asian/Pacific Islander, Native American or other. However, the 27 participating states report race and ethnicity in many different ways. The final KID variable is actually a combined indicator of race and ethnicity, with Hispanic ethnicity taking precedence in final assignment. For example, if a state reports a child's race as white and ethnicity as Hispanic, KID 2000 "race" was coded as Hispanic. All but two KID 2000 states, Iowa and North Carolina, reported Hispanic ethnicity as a separate variable or as a category in a combined race/ethnicity variable.

#### Covariates

Age is an important risk factor for pediatric appendicitis rupture [9,13,14], and hence a necessary covariate, because it is more difficult to quickly diagnose appendicitis in younger children. The literature is inconsistent regarding male gender as a risk factor for rupture. Gadomski et al. [13] found boys to be at elevated risk for a Maryland sample, while Guagliardo et al. [9] and Ponsky et al. [10] did not detect gender differences in larger samples. We include gender as a covariate as a precaution and to further explore it as a risk factor.

ZIP code median annual income was used as a proxy for family income. While less relevant than direct measures of family income or wealth, ZIP code median annual income has been shown to be a useful proxy in other hospital utilization studies [15,16] Guagliardo et al. [9] found lower ZIP code income to be a risk factor for AR, independent of insurance type and other covariates. In KID 2000 this variable is coded in the four categories used for the HCUP National Inpatient Sample [17]: $0–$24,999, $25,000–$34,999, $35,000–$44,000; and ≥ $45,000.

Insurance type (i.e. expected primary payer) is a well-established indicator of barriers to healthcare for most medical conditions, including acute appendicitis and timely appendectomy [9,10,14,15]. Typically, privately insured patients receive the most timely and highest quality care, followed by publicly insured patients, with the uninsured having the most difficulty. We created an expected payer variable that mimics these three categories. KID 2000 codes its uniform primary payer variable as Medicare, Medicaid, private insurance, self-pay, no charge, and other. We grouped Medicare and Medicaid into a combined publicly insured category. We carefully explored the state-specific codes underlying the KID 2000 "other payer" category to see if there were cases in this residual category that could be reasonably assigned to public, private or uninsured categories. This was possible for a worthwhile number of cases. For example, in California several "other government" and indigent care programs designed for the needy were coded as "other payer" in KID 2000. We grouped those with our publicly insured cases. (The authors may be contacted for a complete list of recodes.) Some of the other remaining KID 2000 "other payer" cases were excluded because they could not be confidently assigned to one of our three target categories. However, we created our own "other" category to approximate an uninsured group. It consisted of self-pay, no-charge, charity, and otherwise uninsured cases. We recognize that a very small percentage of these cases could be from uninsured but non-needy families. Therefore, we labeled the group as "other" rather than uninsured.

Admission from an emergency department (ED) appears to be a protective factor against risk of rupture [9,14,15,18], possibly due to quicker diagnosis than for cases who delayed seeking care or were first seen by a primary care provider. We created an admission source variable with three categories – admitted from ED, referred to the hospital for inpatient treatment by any other healthcare provider (e.g. other hospital, within-hospital clinic, stand-alone clinic or HMO), and "other source". The latter includes self-referrals and other unspecified sources.

There is continued interest in the effect of hospital teaching status on outcomes [19]. Teaching hospitals are noted for better outcomes, including appendicitis outcomes [15], but lower patient satisfaction.([20]. Therefore we included hospital teaching status as a covariate.

There is also interest in the effect of hospital patient volume on appendicitis outcome. The idea is that greater experience diagnosing and treating a given condition will improve an institution's performance [21-23]. The number of pediatric discharges from KID 2000 hospitals ranged from 4–22,785. We divided the hospitals into volume quartiles, assigned the quartile value of the discharging hospital to each patient, and used the volume quartile assignment as a covariate in the logistic regressions.

### Analysis

As noted in Guagliardo, et al. [9], most previous studies of AR [13-15,18] found little evidence of race/ethnicity disparities because their regression models overcontrolled for the factors that mediate disparities. Overcontrolling is a common methodological hazard [24]. In order to reveal disparities at different levels of control for mediating factors we developed four logistic regression models. The first model used race/ethnicity alone as a predictor of AR odds. The second model included the biological and developmental covariates, gender and age. This model is of interest because it adjusts for factors that are not amenable to health policy or social policy changes. The disparities revealed by this model should be targeted with policy changes. The third model adds the social and system factors, median ZIP code income, insurance type and admission source. The final and fullest model adds the hospital-level factors, pediatric discharge volume and hospital teaching status. While overcontrolled, we report the fullest model because it is more comparable to previously published studies, and because it bears interesting revelations about the covariates.

All analyses were performed with SAS 9.1.3 [25]. We tested for mulitcollinearity among our variables using the collinearity index (PROC REG with the COLLINOINT option) [26]. Frequencies and means were computed with the SAS SURVEYFREQ and SURVEYMEANS procedures using the appropriate survey weighting variables. Regressions were modeled with PROC SUVEYLOGISTIC, a procedure for weighted logistic regression, taking into account the sampling design and sample discharge weights using the methods outlined in Houchens and Elixhauser [27]. We specified the hospital cluster and survey stratum in the CLUSTER and STRATUM statements. These methods assured unbiased variance estimates.

## Results

There were 40,762 acute appendicitis cases in KID 2000. Our exclusion of cases with problematic race/ethnicity diagnosis codes limited our analyses to 32,784 cases. Weighted, these cases represented approximately 62,555 children hospitalized for acute appendicitis in 2000. All table values are weighted to represent national estimates.

Table 1 compares the number and proportion of appendiceal rupture (AR) and non-rupture outcomes for all study variables. All variables except gender showed statistically significant variations in rates of AR among groups (P < 0.05). Higher AR rates were noted for children who were minorities, younger, from poorer ZIP codes, lacking private insurance, referred from somewhere other than the ED, discharged from a teaching hospital, and discharged from a high-volume hospital.

**Table 1:** National estimates of ruptured and unruptured appendicitis cases for children 4–18 years old. Obtained from the KID Year 2000 data set. (Weighted count of cases = 62,555).

		*Ruptures (%)*	*Non-ruptures (%)*
Race/ethnicity*	white	12,056 (29%)	29,514 (71%)
	black	1,474 (36%)	2,579 (63%)
	Hispanic	5,539 (36%)	1,001 (64%)
	Asian	499 (36%)	893 (64%)

Gender	male	11,947 (31%)	26,247 (69%)
	female	7,613 (31%)	16,726 (69%)

Age group*	4–8	5,387 (42%)	7,591 (58%)
	9–11	4,189 (31%)	9,221 (69%)
	12–14	5,075 (31%)	11,444 (69%)
	15–18	4,918 (25%)	14,731 (75%)

ZIP code median annual household income*	$0–$24,999	2,059 (38%)	3,399 (62%)
	$25,000–$34,999	5,612 (33%)	11,573 (67%)
	$35,000–$44,999	5,284 (30%)	12,097 (70%)
	≥ $45,000	6,309 (29%)	15,198 (71%)

Expected primary payer*	private	12,226 (29%)	29,866 (71%)
	public	5,552 (37%)	9,351 (63%)
	other	1,554 (33%)	3,179 (67%)

Admission source*	emergency department	13,068 (30%)	30,273 (70%)
	other healthcare referral	4,461 (35%)	8,281 (65%)
	other	1,097 (32%)	2,193 (68%)

Hospital type*	teaching	9,552 (35%)	18,114 (65%)
	other	10,016 (29%)	24,836 (71%)

Pediatric discharge volume*	4–1,578	4,759 (28%)	12,474 (72%)
	1,579–3,471	4,343 (28%)	11,213 (72%)
	3,472–6,582	4,320 (32%)	9,568 (68%)
	6,583–22,785	6,045 (38%)	9,733 (62%)

		*Means (95% CIs)*	

Length of stay*		5.5 (5.4–5.6)	2.0 (1.9–2.0)
Total charges*		$17,905 ($17,473–$18,336)	$9,076 ($8,956–$9,198)

*Chi-square p < 0.01.

Utilization measures were much higher for AR cases. Mean length of stay was 5.5 days for AR cases, or 175% higher than the 2.0 day mean for non-ruptured cases. Mean total charges were 97% higher for AR cases, at $17,905 versus $9,076.

Odds of AR are presented in Table 2. In the first model, of unadjusted odds by race/ethnicity categories, all the minority groups have 36%-40% higher odds of rupture compared to whites. These odds decrease but remain significant across the table for all three minority groups even as covariate adjustment factors are added. The second model includes the covariates, gender and age group. Gender is irrelevant to odds of rupture in this and all subsequent models. On the other hand age is meaningful in all models that include it. As expected, younger children are consistently at higher risk of rupture – between 27% and 105% higher depending on the age group and adjustment covariables used. The third model includes social and healthcare system factors. Private insurance and higher income are protective against AR in this as well as the final model. Children referred to the hospital from a non-emergency department healthcare setting had nearly 30% higher odds of AR than children admitted directly from the discharging hospital's ED. This admission source pattern holds for the final, fullest model, which includes hospital characteristics as covariates. In that model teaching and non-teaching hospital discharges are indistinguishable for odds of AR. Surprisingly, lower pediatric discharge volume appears to have been a protective factor against odds of AR. Compared to children treated at hospitals in the highest volume quartile, children discharged from hospitals in the three lower volume groups had 21%-28% better odds of avoiding AR.

**Table 2:** Odds ratios for appendiceal rupture among children in the United States.

		*Unadjusted race/ethnicity odds*	*Race/ethnicity odds adjusted for biologic factors*	*With additional adjustments for social and system factors*	*With additional adjustments for hospital factors*
Race/ethnicity	white	*ref*	*ref*	*ref*	*ref*
	black	1.40 (1.27–1.55)	1.38 (1.25–1.53)	1.27 (1.14–1.42)	1.23 (1.10–1.37)
	Hispanic	1.36 (1.28–1.44)	1.24 (1.17–1.31)	1.14 (1.07–1.22)	1.07 (1.00–1.15)
	Asian	1.37 (1.16–1.61)	1.32 (1.13–1.52)	1.29 (1.10–1.53)	1.24 (1.05–1.46)

Gender	male		1.00 (0.95–1.06)	0.99 (0.94–1.05)	0.99 (0.94–1.05)
	female		*ref*	*ref*	*ref*

Age group	4–8		2.05 (1.90–2.21)	2.02 (1.87–2.18)	1.90 (1.76–2.06)
	9–11		1.36 (1.25–1.47)	1.36 (1.25–1.47)	1.30 (1.20–1.41)
	12–14		1.32 (1.23–1.42)	1.31 (1.22–1.42)	1.28 (1.18–1.38)
	15–18		*ref*	*ref*	*ref*

ZIP code median annual household income	$0–$24,999			1.15 (1.05–1.27)	1.13 (1.02–1.24)
	$25,000–$34,999			1.06 (0.99–1.14)	1.11 (1.03–1.20)
	$35,000–$44,999			1.00 (0.93–1.08)	1.04 (0.96–1.12)
	≥ $45,000			*ref*	*ref*

Expected primary payer	private			*ref*	*ref*
	public			1.10 (0.99–1.23)	1.09 (0.98–1.21)
	other			0.87 (0.78–0.97)	0.87 (0.78–0.96)

Admission source	emergency department			*ref*	*ref*
	other healthcare referral			1.28 (1.19–1.38)	1.29 (1.20–1.39)
	other			1.03 (0.93–1.14)	1.05 (0.95–1.16)

Hospital type	teaching				1.06 (0.99–1.14)
	other				*ref*

Pediatric discharge volume	4–1,578				0.72 (0.65–0.80)
	1,579–3,471				0.73 (0.66–0.80)
	3,472–6,582				0.79 (0.73–0.86)
	6,583–22,785				*ref*

Sample size	unweighted	32,784	32,773	30,940	30,940
	weighted	62,555	62,533	57,791	57,791

## Discussion

Appendiceal rupture (AR) was much more burdensome than appendectomy without rupture. The mean total hospital charges were 97% higher for AR cases, while mean length of hospital stay was 175% longer. These additional burdens fell disproportionately on minority children. Results for the first regression model in Table 2 show unadjusted racial/ethnic disparities in acute appendicitis outcome. However, the second regression model is more relevant to the national goal of reducing disparities, because it includes adjustments for factors that are not amenable to changes in health policy, social policy or medical practice – patient age and gender. Disparities revealed in this model should be targeted for elimination. African American children have approximately 38% greater odds of appendicitis rupture (AR) compared to whites, and the relative odds are not much better for Hispanic or Asian children – 24% and 32% higher, respectively.

These national disparity estimates differ somewhat from findings reported for other samples. Among 1997 California pediatric discharges Guagliardo et al. [9] found that African American and white children had indistinguishable AR rates, while Hispanic and Asian children had higher odds of AR – 45% and 30%, respectively. They found a different pattern of disparities among 1995 New York discharges. There, white and Hispanic children were indistinguishable, while African American children had 47% higher odds and Asian children had 116% higher odds of AR. In a sample of discharges from children's hospitals Ponsky et al. [10] found that African American children had 13% higher odds and Asian children had 66% higher odds of AR compared to white children. However, they could not identify Hispanic children in their sample, and they used different adjustment variables.

In spite of the incomparability of the samples studied to date, a pattern is emerging. White children have never been found to be at greater odds of AR than any minority group, while minority children are usually found to have poorer appendicitis outcomes than white children. The key question remains. Why is there racial/ethnic disparity in acute appendicitis outcomes?

To explore this question it is helpful to view acute appendicitis as a "delay-sensitive" condition [28]. Once its clock starts then rupture, broader infection, bleeding and death are inevitable without surgery. Lacking evidence to the contrary, it is generally assumed that the disease progresses at the same average rate for all social groups. Therefore inter-group differences in average delay of key milestones in the disease course must account for the disparities. The milestones include first complaint of abdominal pain, parental recognition of urgency, initial seeking of professional care, performance of diagnostic procedures and/or referrals to other healthcare facilities, eventual correct diagnosis, and finally surgical intervention. Reductions in time between any of these milestones will reduce the chance of rupture. Research suggests that in the U.S. there is little or no delay between correct diagnosis and surgery [12]. Children get to the operating room quickly once the diagnosis is made. Therefore, the disparities we have discovered are probably due to longer average delays for minorities prior to correct diagnosis.

Factors that can delay care seeking and timely diagnosis include family health beliefs and economic condition, insurance coverage, physician quality and distance to healthcare provider. We expected that our fuller regression models would provide insight into the effects of some of these factors. Two of the covariates used are taken from major domains generally involved in the production of racial/ethnic disparities in health and healthcare – income [29] and insurance type [30]. As mediators of disparity such as these are added to regression models, apparent race/ethnicity differences should decrease and eventually disappear if all explanatory factors could be included. Yet our fullest model in Table 2 has not achieved this ideal. In it all minority groups still have higher odds of AR relative to white children. This could be because our covariables are imperfect representatives of their domains. For example, insurance type may be too coarse of a measure of insurance quality, e.g. privately insured white children were in better plans than privately insured minority children. It is also possible that administratively derived data sets such as KID 2000 do not contain proxies for important disparity-producing factors. Two prime examples are language and cultural differences between patients and their healthcare providers [31,32], and level of geographic availability of local care providers [33]. Including more and better covariables might have accounted for all race/ethnic disparities. Furthermore, the behavior of these covariables in regression models could point out socioeconomic, demographic and health services factors to be targeted to achieve better outcomes and less disparity. However, finding a fully explanatory model could not excuse the disparities revealed in our second model (Table 2). The disparities revealed in that model should remain as the national indicators of disparity and should be targeted for reduction [34].

The disparity differences found between California, New York and the current national sample suggest that local sociocultural factors are at play. Guagliardo et al. [9] suggested a link between odds of being foreign-born in the two states and odds of AR. They hypothesized that general degree of acculturation could be a major precipitator of disparities, acting through language barriers, preferences for traditional healing and concern among undocumented immigrants for engaging the healthcare system.

A definitive understanding of the causes of disparity in pediatric AR rates will require prospective, primary data collection, including family interviews and in depth case reviews with special attention to the timing of the aforementioned milestones to discover the social, economic, provider and health systems circumstances associated with delayed surgery. However, that the precise causes of disparities remain unclear should not detract from the major thrust of this report. The disparities are real, occur on a national scale, and are most likely due to socioeconomic, cultural and healthcare system factors.

While the covariates in the fuller models do not account for all of the disparities and are not the main focus of this study, their effects are nonetheless noteworthy. The negative effect of lower income is consistent with a large literature on income, wealth and health [35,36]. As found in previous studies [9,14,15,18], admission from the emergency department (ED) of the hospital that performed the appendectomy reduces the odds of AR. In contrast, patients who go to other healthcare settings before referral to the surgical facility tend to have poorer outcomes, ostensibly due to the additional delay. There are similar findings in the acute myocardial infarction and stroke literature [37]. Patients with chest pains or stroke symptoms should go immediately to the nearest ED. Yet it would be premature to recommend that all children with abdominal pain report immediately to the nearest ED. Abdominal pain has many causes [8], and such a recommendation would fly in the face of decades of efforts to reduce unnecessary ED utilization [38]. Careful and thorough research is required to developed optimal recommendations to reduce both AR rates and unnecessary ED utilization [38-40].

Teaching hospitals have a reputation for lower patient satisfaction but better medical outcomes than non-teaching hospitals [19,20,41,42]. Findings for AR have been mixed. Braveman et al. [15] reported better outcomes for adults discharged from teaching hospitals, while Guagliardo et al. [9] found poorer outcomes for children discharged from teaching hospitals. Here, adjusted odds of AR are estimated to be 6% higher for children discharged from teaching hospitals, although the difference is not statistically significant. Still, future AR studies should consider hospital teaching status as a covariable.

Of all the covariables, pediatric discharge volume yielded the most surprising results. Contrary to the "practice makes perfect" maxim [21], neither of the previous large-sample pediatric studies [9,10] found a relationship between volume and outcome. The current analysis actually showed the reverse effect. Hospitals in the highest volume group had significantly higher AR rates. This is difficult to explain. It is possible that high-volume hospitals have unfavorable staff-to-patient ratios, leading to additional delays in care. It might also be that these hospitals are located in underserved areas, and hence more of their patients must travel farther for service, which might delay diagnosis. We know of no published studies that test these hypotheses. It is interesting to note that Smink et al. [22] found significantly higher rates of *negative *appendectomy in low-volume hospitals. (A negative appendectomy is an unnecessary surgery resulting from an incorrect diagnosis.) Smink et al. analyzed the 1997 version of KID. Comparing their nationally representative results with ours, it appears that two kinds of error might be in effect. Low-volume facilities might have excessive rates of misdiagnosis and premature surgery, while high-volume facilities might have excessive rates of delayed diagnosis and delayed surgery. We are planning another study to explore these questions. Recommendations are not possible at this time.

## Additional limitations

Nearly 20% of otherwise eligible KID cases were missing race/ethnicity or were in the American Indian or "other" categories. Thus they were not used in the analyses. Fortunately these cases did not differ from our sample in the rate of AR (30% versus 31%, p < 0.52), reducing the probability that our analyses are biased. However, the problem could become acute if the proportion of missing race/ethnicity cases increases in future releases of KID. This underscores the danger of initiatives to prevent collection of race/ethnicity data in healthcare administrative databases, such as California's Proposition 54 [43].

Hospital ownership (public versus private) is often considered as a covariable in administrative data studies. However, too many states in the KID 2000 system reported ownership with a combined "public or private" categorization, making the distinction impossible in the current analysis. We do not consider this a serious limitation, as a previous study showed ownership not to be a factor for risk of pediatric appendicitis rupture [9].

It is very common in the healthcare literature to use ZIP code median income as a proxy for individual income as we have done. However, there is always the risk of ecological fallacy – the bias that can result from using aggregate data in lieu of individual-level measurements [44]. Studies have shown that aggregate statistics from the census block group and census tract levels are useful proxies for individual-level measures [45]. However, ZIP code socioeconomic indicators are somewhat insensitive to geographic variation in health indicators [46]. While this might diminish the predictive power of our income indicator, we can think of no reason that it would be biased.

## Conclusion

The first National Healthcare Disparities Report states that, "While consistency of measures from year to year is highly desirable, the measures selected for inclusion in the first NHDR represent a small subset of currently available measures and are expected to evolve as the field of health care measurement itself evolves." [4] This paper proposes a new measure for inclusion in future reports – rate of appendiceal rupture (AR). Minority children with acute appendicitis in the U.S. are 24%-38% more likely than white children to experience (AR) and its attendant complications and expenses. Because AR can be avoided with timely surgery, and because rate of progression of infection is not linked to cultural factors, these outcome disparities are probably due to differences in timely access to quality care. Some of the factors that play a role in AR may be amenable to healthcare and social policy changes. It would be worthwhile to attempt to reduce disparities associated with insurance differences, income differences, referral patterns and/or how families seek urgent care, and in-hospital practice differences. However, there are significant residual disparities not attributable to these factors. Further research is required to discover and address the causes.

Regardless of the causes, the disparities are real and significant. It should be a national goal to reduce or eliminate disparities in risk of AR. Analysis of KID data for pediatric acute appendicitis outcomes is relatively straightforward. Therefore, Congressional funding for the survey should continue, and KID-based disparities in AR rates should be included and tracked over time as an indicator of racial/ethnic disparities in the National Healthcare Disparities Report.

## List of abbreviations

AHRQ – Agency for Healthcare Research and Quality

AR – appendicitis rupture, appendiceal rupture

HCUP – Healthcare Cost and Utilization Project

KID – Kids Inpatient Database

NHDR – National Healthcare Disparities Report

## Competing interests

The author(s) declare that they have no competing interests.

## Authors' contributions

Both authors contributed equally to writing the introduction, discussion and conclusions. KAJ obtained the data, performed all statistical analyses, and wrote the methods and results sections. MFG conceptualized the project and performed most of the literature search.
